# Comparative Researches of Semen Arecae and Charred Semen Arecae on Gastrointestinal Motility, Motilin, Substance P, and CCK in Chronically Stressed Rats

**DOI:** 10.1155/2017/1273561

**Published:** 2017-12-11

**Authors:** Sanyin Zhang, Peng Yang, Xixiong Li, Xin Wang, Jiage Song, Wei Peng, Chunjie Wu

**Affiliations:** ^1^Chengdu University of Traditional Chinese Medicine, Chengdu 610075, China; ^2^The Fifth People's Hospital of Chengdu, Chengdu, China

## Abstract

**Aims:**

To compare the effects of Semen Arecae (SA) and Charred Semen Arecae (CSA) on gastrointestinal motility, motilin, substance P (SP), and cholecystokinin (CCK) in chronically stressed rats.

**Methods:**

Rats were randomly divided into control group and stress group. Rats in stress group were randomly exposed to a variety of unpredictable stimulations for 21 days. Then, the rats were treated orally with distilled water, SA, CSA, and mosapride for 7 days. Gastric residue rate and intestinal propulsion rate were evaluated. Serum levels of motilin and SP were measured by enzyme-linked immunosorbent assay (ELISA). CCK mRNA was quantified by using quantitative real-time PCR (qRT-PCR).

**Results:**

Both SA and CSA improved the intestinal propulsion and reduced the gastric residue in chronically stressed rats. Furthermore, the serum levels of motilin and SP were significantly higher and the CCK mRNA expressions in intestine and hypothalamus were downregulated in SA and CSA groups. Furthermore, it was found that CSA is more effective.

**Conclusion:**

Both SA and CSA enhanced gastrointestinal motility and increased serum levels of motilin and SP in chronically stressed rats via downregulating CCK mRNA expressions in intestine and hypothalamus. Importantly, CSA possessed more effective promoting effects.

## 1. Introduction

Functional dyspepsia (FD), a common functional gastrointestinal disorder, is dyspepsia without obvious organic disease [1]. Previous survey revealed that its prevalence is 25% in the United States, 12%–25% in China, and 23.8% in the United Kingdom [2–4]. Based on the Rome III criteria, bloating, belching, early satiety, abdominal distension, nausea, or indigestion during the last three months with symptom onset at least six months ago may be symptoms of FD [5]. FD is classified into postprandial distress syndrome (PDS) and epigastric pain syndrome (EPS); however, these two syndromes above may overlap [6]. Life quality of FD patient would be highly decreased due to the symptoms of FD, especially the abdominal pain and indigestion [7].

So far, the pathogenesis of FD remains unknown but is involved in many factors, including impairment of gastric motility, visceral hypersensitivity, moderate inflammatory reactions of duodenal mucosa, and psychosocial disorders [8]. Currently, increasing evidences have indicated that gastrointestinal motor dysfunction might be a crucial pathogenesis of FD [9]. In the 1960s and 1970s, the term “gut-brain axis” was coined after the finding of some peptides in both brain and gastrointestinal tract, which refers to the bidirectional communication between the gut and the brain [10]. Gut-brain peptide such as motilin and substance P (SP) plays important roles in the bidirectional gut-brain communication.

Importantly, gastrointestinal motility is regulated by many brain-gut peptides. Motilin, a 22-amino acid residue polypeptide secreted by endocrine M cells, has a powerful fundic pouch motor-stimulating activity [11, 12], and SP, a member of tachykinin family of neuropeptides widely distributed throughout the central nervous system (CNS), controls the enteric noncholinergic motor inhibition of the fundus [13].

Areca nut, commonly named as Semen Arecae (SA), is the seeds of* Areca catechu* L. and has been used as an important Chinese herbal medicine for thousands of years [14]. SA is commonly processed as charred SA (CSA), and both SA and CSA can improve effects on gastrointestinal motor function [15–17]. In particular, CSA has been characterized for its improving effects on gastrointestinal motor function and used to alleviate various symptoms of dyspepsia [15]. However, so far, it is still unclear that the pharmacological mechanism of SA and CSA on gastrointestinal motor function and whether CSA is more effective than SA for treating gastrointestinal dysfunctions (such as functional dyspepsia) or not. Thus, in our present study, we investigated the effects of SA and CSA on gastrointestinal motility in a chronically stressed rat model and subsequently measured the serum levels of motilin and SP and determined the mRNA expressions of CCK in intestine and hypothalamus.

## 2. Material and Methods

### 2.1. Drugs

In this research, the raw materials of SA were obtained from Sichuan Neautus Traditional Chinese Medicine Co., Ltd. (Chengdu, China), and CSA were processed by the Key Laboratory of Technology of Chinese Medicine Processing, Chengdu University of Traditional Chinese Medicine (Chengdu, China). Mosapride citrate was purchased from the Yabao Pharmaceutical Co. (Yuncheng, China) and dissolved in distilled water to a final concentration of 0.25 mg/mL. SA (3 g/kg), CSA (3 g/kg), and Mosapride (2.5 mg/kg) were administered orally to rats treated by SA, CSA, and Mosapride groups consecutively for 7 days, respectively.

### 2.2. Animals

Male Sprague-Dawley (SD) rats (7 weeks of age, 200–230 g) were used in the experiments. Animals were housed in groups of five per cage and housed with a regular light-dark cycle (lights on at 7 am, lights off at 7 pm) at a controlled temperature (25°C ±1°C). They were provided with a standard pellet diet and given water ad libitum. All rats were purchased from Biotechnology Co., Ltd. of DaShuo (Chengdu, China).

Sixty rats were randomly divided into control group (*n* = 10) and stress group. After the rats in stress group were exposed to chronic stressors for 21 days, ten rats were randomly selected as a pathology group. Besides, the other forty rats were randomly divided into model group (M), SA group, CSA group, and Mosapride group (*n* = 10, each group consisted of 5 female and 5 male rats). The experiments were carried out in accordance with the guidelines of the Management Committee for Experimental Animals and approved by the Ethics Committee of Chengdu University of TCM (Chengdu, China).

### 2.3. Rat Model of Chronic Stress 

Rats in stress groups were randomly exposed to different chronic stressors (including eight different kinds of stress), as previously reported [18, 19], daily from day 1 to day 21 as follows: food deprivation (24 hours), water deprivation (24 hours), tail pinching (2 cm apart from the end of the tail) for 1 min, hung upside-down for 5 min, cage tilting for 24 hours (45°C), restraining in a rat fixation device for 2 hours, day and night reversal, and cold stimulation at 4°C for 5 min. The rats were subjected to one of these stressors at a different time each day and the same stressor was not applied consecutively on 2 days so the animals could not predict the occurrence of any stimulation. The body weights of rats in control and stress groups were recorded regularly during the experiments on days 0, 7, 14, and 21.

### 2.4. Histopathological Examination

After 21 days' modeling, ten rats in pathology group were killed. And stomach and small intestine were rapidly removed from the ten rats. Stomach and small intestine were opened to observe whether the gastric and small intestine mucous were normal and smooth, congestion, edema, and erosion. Stomach and small intestine were subjected to histological examination. They were pressed in a fixation medium of 10% solution of buffered formalin. The next steps were followed by dehydration and then enclosed in paraffin. The sections were sectioned (specimens were cut into 7 *μ*m thick sections) and stained with hematoxylin and eosin (H&E) prior to microscopic examination and observed under an Olympus microscope (Olympus, Tokyo, Japan).

### 2.5. Detection of Gastric Residue and Intestinal Propulsion

Gastric residue and intestinal propulsion determinations were carried to evaluate the gastric motility according to the reported methods with minor modifications [20]. After six days' drug treatments, rats were fasted for 24 h and then administered the last drug and water. One hour later after the last drug, all rats were given 2 mL semisolid paste. Twenty minutes later, the rats were sacrificed, and the rate of semisolid paste in the stomach and the rate of semisolid paste propulsion in the small intestine were measured. The stomach was exposed by laparotomy and removed. Then, the percentage traverse of the charcoal meal in the small intestine was determined. The gastric residue rate and intestinal propulsion rate for each rat was calculated according to the following formula: gastric residue rate (%) = gastric residue quality/semisolid paste quality × 100%, and intestinal propulsion rate (%) = advanced length of black semisolid paste/total length of the small intestine × 100%.

### 2.6. Samples Collection

Blood samples were collected from the heart of rats after anesthesia. Serum separator tubes were used to collect and allowed to clot for 2 hours at room temperature. Serum was removed and stored at −80°C after the blood has been centrifuged. Intestine and hypothalamus were immediately removed from an environment with a low temperature at stored in liquid nitrogen.

### 2.7. Measurement of Serum Levels of Motilin and SP

The frozen serum was brought to room temperature slowly and mixed gently. Then, serum levels of motilin and SP were measured by ELISA according to the instructions of motilin ELISA kit (Shanghai Blue Gene Biotech Co., Shanghai, China) and SP ELISA kit (Cayman Chemical Co., Michigan, USA), respectively.

### 2.8. Expression Level of CCK mRNA in Intestine and Hypothalamus

The mRNA expressions of CCK in intestine and hypothalamus were quantified by using quantitative real-time PCR (qRT-PCR). Total RNA was extracted from the rats' hypothalamus and intestine by using Trizol reagent (Invitrogen, USA), and total 2 *μ*g RNA was reverse transcribed by using Promega GoScript (Promega, USA). RNA concentration was measured by 1.5% agarose gel electrophoresis. Primers were designed by Invitrogen Biotech Co. using Primer-BLAST and the primers of the CCK (NM_012829.2) gene were 5′-ATGAAGTGCGGCGTGTGTCT-3′ (Forward) and 5′-GGGTCCACAGCTTCTACAGGG-3′ (Reverse) (92 bp), respectively, and primers of *β*-actin (NM_021669.2, used as the internal control), were 5′-ACCAGAAAGCCCAGCAGAGAA-3′ (Forward) and 5′-GAAGGGAGCATTGAACCTGATT-3′ (Reverse) (146 bp), respectively. The PCR products were run on 1.5% agarose gel to confirm that these products were of the expected size.

### 2.9. Statistical Analysis

Data are expressed as mean ± SD and were analyzed statistically using SPSS 17.0 software (SPSS, Chicago, IL, USA). Statistical analysis was performed by one-way analysis of variance (ANOVA), LSD test, or rank sum test. *P* < 0.05 was considered statistically significant.

## 3. Results

### 3.1. Body Weight Changes during Restraint Stress Period

**Figure 1:**
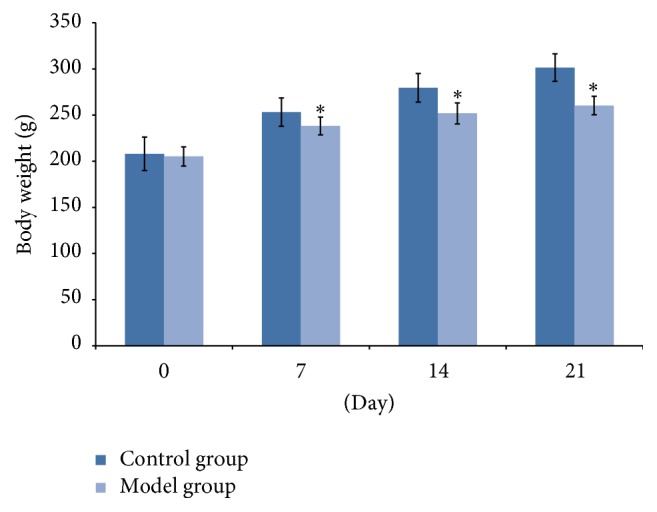
Rat in model randomly exposed to different chronic stressors from day 1 to day 21. And the body weight was lower in model group compared with control group on days 0, 7, 14, and 21. ^*∗*^*P* < 0.05 compared with the control group. Data were presented as mean ± SD, analyzed by one-way ANOVA, and followed by LSD test or rank sum test.

As shown in Figure 1, no significant difference in weight was observed between control group and stress group at beginning of the study. Furthermore, changes in body weight were measured during the stress period. After 7, 14, and 21 days' test, rats' body weights were significantly decreased in the model group compared with the control group (*P* < 0.05).

### 3.2. Pathological Examination for Pathology Group Rats after 21 Days' Modeling

**Figure 2:**
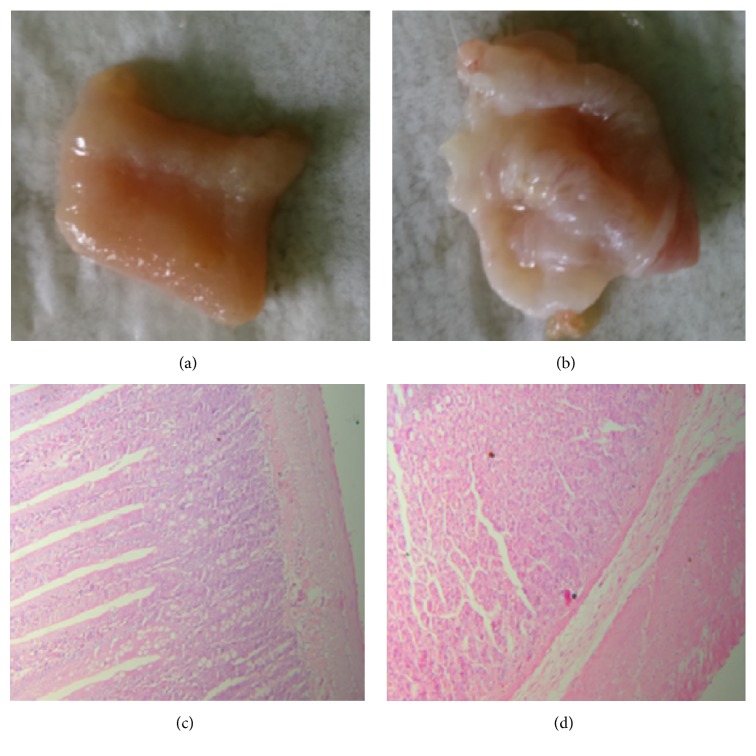
Results of the anatomical and pathological examinations. (a) and (b) represented the shapes of small intestine and stomach, respectively, and (c) and (d) represented the tissue sections of small intestine and stomach, respectively.

Rats were euthanized and anatomized after 21 days' modeling. For the model rats, the tissues have normal color, and no congestion, edema, erosion, ulcer, and swelling were observed (Figures 2(a) and 2(b)). In addition, pathological examinations results also showed that tissues structure of the gastric and small intestine mucosa were normal, and no obvious fibroplasia, hemangiectasis, and inflammatory cell infiltration were observed (Figures 2(c) and 2(d)).

### 3.3. Gastric Residue and Intestinal Propulsion Rates for Chronically Stressed Rats

As shown in Table 1, effects of SA and CSA on gastric residue and intestinal propulsion rates in chronically stressed rats were described. The present study demonstrated that the gastric residue rate was significantly increased in chronically stressed rats (*P* < 0.01), whereas the intestinal propulsion rate was decreased (*P* < 0.05), compared with control rats. After treatment with SA and CSA, the gastric residue rates were significantly decreased (*P* < 0.01), whereas the intestinal propulsion rates were notably increased (*P* < 0.01), compared with model rats. Importantly, the gastric residue rates of CSA treated rats were lower than that of SA treated rats (*P* < 0.05), whereas the intestinal propulsion rates were higher than that of SA rats (*P* < 0.05).

### 3.4. Serum Levels of Motilin and SP in Chronically Stressed Rats

**Figure 3:**
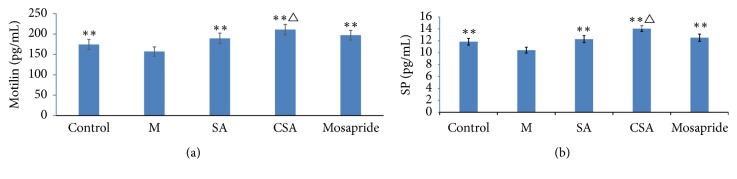
Serum levels of motilin (a) and SP (b) in chronically stressed rats. ^*∗∗*^*P* < 0.01 compared with the model group; ^△^*P* < 0.05 compared with the SA group. Data were presented as mean ± SD, analyzed by one-way ANOVA, and followed by LSD test or rank sum test.

Compared to control mice, serum levels of motilin and SP of model rat were reduced model group (*P* < 0.01). Interestingly, serum levels of motilin and SP of the rats treated with SA, CSA, and Mosapride were also significantly increased compared to model rats (*P* < 0.01, Figure 3). Besides, serum levels of motilin and SP in CSA treated rats were significantly higher than that of SA treated rats (*P* < 0.05, Figure 3).

### 3.5. CCK mRNA Expression in Intestine and Hypothalamus 

**Figure 4:**
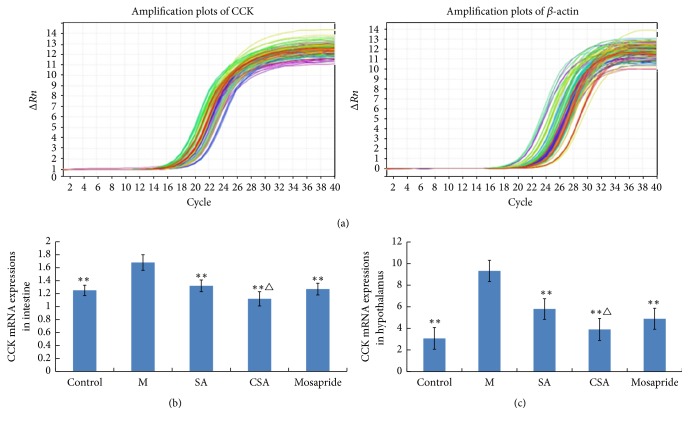
Amplification plots (a) and CCK mRNA expressions in intestine (b) and hypothalamus (c). ^*∗∗*^*P* < 0.01 compared with model group (M); ^△^*P* < 0.05 compared with SA group. Data were presented as mean ± SD, analyzed by one-way ANOVA, and followed by LSD test or rank sum test.

**Figure 5:**
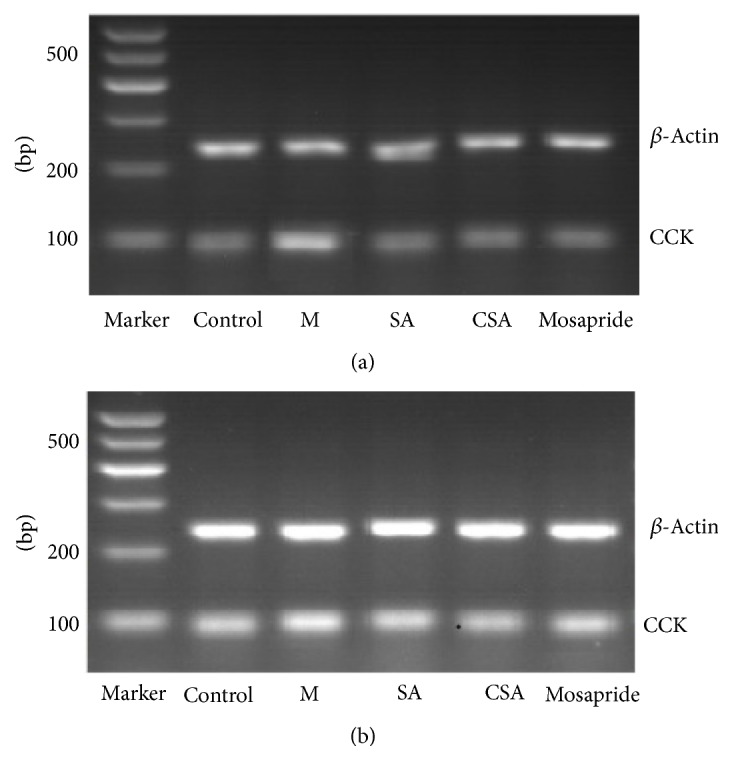
Gel electrophoresis results of the intestine (a) and hypothalamus (b) qRT-PCR products on 3% agarose gel.

As can be seen from Figure 4, the CCK mRNA expressions in small intestine and hypothalamus in control rats were significantly higher than that of model rats (*P* < 0.01, Figure 4). The CCK mRNA expressions in small intestine and hypothalamus were significantly lower in SA, CA, and Mosapride groups compared to model rats (*P* < 0.01, Figure 4). Importantly, the mRNA expressions of CCK in small intestine and hypothalamus of CSA treated rats were significantly lower than that of SA treated rats (*P* < 0.01, Figures 4(b) and 4(c)). In addition, the gel electrophoresis results also confirmed the above qRT-PCR results (Figure 5).

## 4. Discussion

FD is involved in complex pathogenetic mechanisms [21], including motility disorders, visceral hypersensitivity, acid disorders,* Helicobacter pylori* infection, or psychosocial factors [22, 23]. As many people suffering from FD, a typical consequence of this disease is a reduced quality of life, which in turn has a significant economic impact on the healthcare system [7, 24]. Both 5-HT_4_ receptor agonists (Mosapride citrate, cisapride monohydrate, and tegaserod) and dopamine 2 (D_2_) receptor antagonists (domperidone and itopride hydrochloride) are currently used to treat FD patients in some countries. However, pharmacological therapy by these drugs for FD patients has resulted in outcomes that have been unsatisfactory [25, 26]. Therefore, finding more effective treatment strategies for treating FD is necessary.

Stressed animal is often used as a model of FD, which have been reported to affect different parameters influencing gastric functions [27]. In the present study, rats were exposed to severe different chronic stressors. A significant difference in body weight due to chronically stressed process was also observed between control and model group. All rats in pathology group showed normal structure and no lesions were observed after 21 days' modeling, which indicated no evidence of structural disease. What is more, the gastrointestinal motility was lower in chronically stressed rats than in normal control rats, which is consistent with the reported findings [28]. All these data indicate that the chronically stressed rat model was successfully created by chronic stressors.

Currently, effect of digestive system is recognized as the characteristic pharmacological activity of SA and has been comprehensively investigated [29–31]. Our present results also showed that both SA and CSA have significant promoting effects on gastrointestinal motility in chronically stressed rats. So far, more than 59 compounds have been isolated from SA, and pyridine-type alkaloids and condensed tannins have been identified as its characteristic constituents [15]. It has been reported that total alkaloids content of SA is approximately 0.3–0.7% [32], mainly arecoline, arecaidine, guvacoline, and guvacine. Alkaloids in SA are recognized as the effective components to improve the gastrointestinal function [15, 33, 34]. Arecoline can significantly increase gastrointestinal motility in rabbits and in rats* in vivo* via stimulation of the M3 receptor and the verapamil-sensitive Ca^2+^ channel [34–36]. In our previous research, the promoting effect of SA on small intestine could be reduced by removing the alkaloids, indicating that the alkaloids play major role in the promoting effect of SA on gastrointestinal smooth muscle [37]. After processing, the contents of alkaloids especially arecoline decreased sharply [15, 38, 39]. In this study, we also find that the contents of alkaloids in CSA are significantly lower than in SA (see Figure S1 and Table S1 in Supplementary Material available online at https://doi.org/10.1155/2017/1273561); however, the promoting effects of CSA on gastrointestinal motility were higher than that of SA. Thus, we guess that, besides alkaloids, there are other constituents that play important roles in its promoting effects on gastrointestinal motility. It is reported that Maillard reaction universally exists in heating procedure of food and herb medicine, and it is the main course leading to the changes of color and flavor of the object [40, 41]. Previous reports revealed that contents of 5-hydroxymethyl furfural (5-HMF) in SA could be increased highly after processing [42]. 5-HMF, which is a key intermediate of Maillard reaction, has been evaluated as indicator of the severity of heat treatment or length of storage in several heating products [43, 44]. Therefore, 5-HMF and other Maillard reaction produced constituents in CSA maybe responding for its increased promoting effects on gastrointestinal motility.

Motilin induces smooth muscle contraction and improves peristalsis in the small intestine [45, 46] and plays an important role on gastric emptying [47]. It is reported that motilin content is lower in FD patients than in healthy people [48]. In the present study, we found that the serum level of motilin is lower in model rat than that of control rat, and both SA and CSA increased the serum motilin levels. Furthermore, the serum motilin levels in CSA treated rats were significantly higher than that of SA treated rats. Neuropeptides, such as an established brain-gut peptide SP, play important roles in the bidirectional gut-brain communication [10]. It was reported that SP can increase gastrointestinal motility and promote contraction of alimentary tract smooth muscle. AS a major excitatory noncholinergic neurotransmitter, SP can depolarize the membrane potential and thus induces contraction in gastrointestinal smooth muscle [49]. Experimental evidences showed that SP increases visceral sensitivity and accelerates gastrointestinal motility [50, 51]. The present study showed that serum level of SP in model group is lower than that of control group, and both SA and CSA can increase the serum SP levels. Furthermore, similar to the motilin, the serum level of SP in CSA is significantly higher than that of SA group.

CCK belongs to the gut-brain family of peptide hormones and is widely distributed in both the enteric and central nervous systems [52]. This hormone is produced by I-cells and exists in mucosa and circulation in molecular forms [53]. In a previous study, CCK can inhibit gastric motility and emptying [54], and it can also inhibit food intake [55]. It is reported that the CCK levels are higher in FD patients than in normal patients [56], and intravenous injection of CCK can suppresses hunger and feeding in healthy humans [57]. In the present study, the CCK mRNA expressions were increased in not only the intestine but also the hypothalamus of chronically stressed rats. Importantly, for the chronically stressed rats, CCK mRNA expressions in hypothalamus and intestine were lower in CSA treated rats than that of SA rats.

## 5. Conclusion

Our results demonstrated that both SA and CSA can enhance the gastrointestinal motility in chronically stressed rats via increasing the serum levels of motilin and SP and downregulating the CCK expressions in intestine and hypothalamus. In addition, our results also showed that CSA possessed better treatment effects on FD than SA. Our present study provides a pharmacological reference for clinical application of SA and CSA in treatment of FD.

## Supplementary Material

The contents of arecoline, arecaidine and guvacine in SA and CSA.

## Figures and Tables

**Table 1:** Effects of SA and CSA on gastric residue and intestinal propulsion rates in chronically stressed rats.

Groups	*n*	Gastric residue rate/%	Intestinal propulsion rate/%
Control group	10	57.7 ± 2.6^*∗∗*^	61.5 ± 1.9^*∗*^
Model group	10	82.6 ± 1.9	54.9 ± 1.9
SA group	10	71.9 ± 1.7^*∗∗*^	63.2 ± 2.9^*∗∗*^
CSA group	10	62.2 ± 2.3^*∗∗*△^	71.1 ± 1.7^*∗∗*△^
Mosapride group	10	47.8 ± 4.8^*∗∗*^	69.2 ± 1.8^*∗∗*^

^*∗*^ *P* < 0.05, ^*∗∗*^*P* < 0.01, compared with the model group; ^△^*P* < 0.05 compared with the SA group. Data were presented as mean ± SD, analyzed by one-way ANOVA, and followed by LSD test or rank sum test.
