# Supplementary material for: Comparative Researches of Semen Arecae and Charred Semen Arecae on Gastrointestinal Motility, Motilin, Substance P, and CCK in Chronically Stressed Rats
Source: Evid Based Complement Alternat Med. 2017 Dec 11;2017:1273561. doi: 10.1155/2017/1273561 (PMC5742499; doi:10.1155/2017/1273561)
Supplement: Supplementary file 1 — The contents of arecoline, arecaidine and guvacine in SA and CSA. [file 1273561.f1.docx]

**HPLC assay of alkaloids contents in SA and CSA**

The SA or CSA was powdered, and approximately 0.3 g powder was accurately weighed and put into a conical flask. A volume of 15 mL carbonate buffer solution (1.91g sodium carbonate and 0.56g sodium bicarbonate dissolved in 100 mL water) was added to extract the areca nut by reflux (each extraction lasts 30min). The extracts were filtrated using 0.22 μm filtration membrane to obtain the testing samples. Standard agents were dissolved with mobile phase as the standard samples.

The HPLC analysis was performed using a Nucleosil SA SCX (250 mm × 4.6 mm, 5um) column at 30 °C. The mobile phase was composed of acetonitrile (A) - 0.2% phosphoric acid-water solution (pH was adjusted to 3.8 by ammonium hydroxide) (B) (55:45) with a mobile flow rate of 1.0 mL/min. The detection wavelength was at 215 nm with the sample injection volume of 10.0 μL.

**Results of the alkaloids contents in SA and CSA determination by HPLC assay**

The contents of arecoline, arecaidine and guvacine in SA were determined and the results were shown in Table 1 & Figure 1. The results revealed that the contents of arecoline, arecaidine and guvacine in SA & CSA were 4.2 vs. 2.5, 1.3 vs. 0.3 and 0.7 vs. 0.2 mg/g, respectively. The results above indicated that the alkaloids of SA (including arecoline, arecaidine and guvacine) could be significantly decreased by processing.

Table 1. The calibration equation, coefficient of association (R), linearity range and contents of arecoline, arecaidine and guvacine.

|  | Calibration equation | *R* | Linearity range  (μg/mL) | Content (mg/g) | |
| --- | --- | --- | --- | --- | --- |
|  |  |  |  | SA | CSA |
| Arecoline | *Y* = 99105 + 5*X* + 3097 | 0.9997 | 0.1960 - 1.960 | 4.2 | 2.5 |
| Arecaidine | *Y* = 15083 + 6*X* - 2252 | 0.9999 | 0.0484 - 0.484 | 1.3 | 0.3 |
| Guvacine | *Y* = 12135 + 6*X* + 432 | 0.9998 | 0.0484 - 0.484 | 0.7 | 0.2 |

**
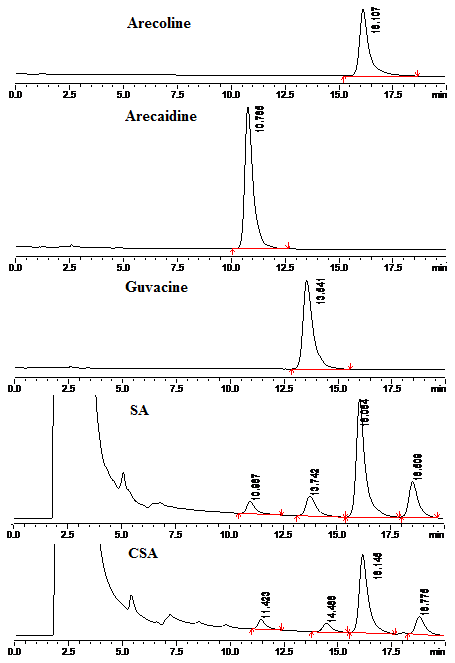
**

Figure **1**. The HPLC chromatograms of standard agents and the SA and CSA.
